# Development and Evaluation of Models for the Relationship between Tree Height and Diameter at Breast Height for Chinese-Fir Plantations in Subtropical China

**DOI:** 10.1371/journal.pone.0125118

**Published:** 2015-04-23

**Authors:** Yan-qiong Li, Xiang-wen Deng, Zhi-hong Huang, Wen-hua Xiang, Wen-de Yan, Pi-feng Lei, Xiao-lu Zhou, Chang-hui Peng

**Affiliations:** 1 Faculty of Life Science and Technology, Central South University of Forestry and Technology, Changsha, Hunan, 410004, China; 2 National Engineering Laboratory for Applied Technology of Forestry & Ecology in South China, Changsha, Hunan, 410004, China; 3 Institute of Environment Sciences, Department of Biological Sciences, University of Quebec at Montreal, Montreal, QC, H3C 3P8, Canada; Chinese Academy of Sciences, CHINA

## Abstract

Tree diameter at breast height (dbh) and height are the most important variables used in forest inventory and management as well as forest carbon-stock estimation. In order to identify the key stand variables that influence the tree height-dbh relationship and to develop and validate a suit of models for predicting tree height, data from 5961 tree samples aged from 6 years to 53 years and collected from 80 Chinese-fir plantation plots were used to fit 39 models, including 33 nonlinear models and 6 linear models, were developed and evaluated into two groups. The results showed that composite models performed better in height estimate than one-independent-variable models. Nonlinear composite Model 34 and linear composite Model 6 were recommended for predicting tree height in Chinese fir plantations with a dbh range between 4 cm and 40 cm when the dbh data for each tree and the quadratic mean dbh of the stand (Dq) and mean height of the stand (Hm) were available. Moreover, Hm could be estimated by using the formula Hm=11.707×ln(Dq)-18.032. Clearly, Dq was the primary stand variable that influenced the height-dbh relationship. The parameters of the models varied according to stand age and site. The inappropriate application of provincial or regional height-dbh models for predicting small tree height at local scale may result in larger uncertainties. The method and the recommended models developed in this study were statistically reliable for applications in growth and yield estimation for even-aged Chinese-fir plantation in Huitong and Changsha. The models could be extended to other regions and to other tree species only after verification in subtropical China.

## Introduction

Tree height and tree diameter at breast height (dbh) are the principal important variables in tree growth models. However, tree height cannot be measured easily in the field. Usually, compared to dbh, the measurement tends to be affected by observer error and is hindered by visual obstructions [1–2]. Tree height and dbh are allometrically related, and the allometric relationship between them is valuable and is commonly used in stand-level planning for silviculture alternatives and effectiveness monitoring [3–4]. Thus, accurate prediction of tree heights is critical in forest inventory compilation, yield modelling, and management decision-making [5–7], as well as carbon budget. Bias in estimating tree height—dbh relationships may result in large uncertainties of estimation for above-ground carbon stocks [8]. Reliable estimates of tree height are essential for assessing above-ground biomass, and strongly affects both ecological and ecophysiological processed-based models of forest growth. In addition, determinations of forest stand biomass have usually been considered to ensure sustainable management, and foresters have applied different methods to obtain such estimates [4]. In evenly-aged, simply structured monocultures, stand-based approaches are appropriate to explain and model tree growth [9]. Individual-tree growth models are fundamental components of forest growth and yield prediction frameworks [10]. Previous ecological research has focused on the height-dbh model building only based on dbh, stand density and basic trunk area. However, model selection and the relationship among stand variables have been less widely studied.

In an earlier study [11], none of the available or derived bivariate generalisations of the univariate lognormal, gamma, and Weibull distributions provided reasonable height-dbh relationships. Several bivariate distributions, potentially useful for describing the joint frequency distribution of tree diameters and heights in even-aged stands of timber, were reviewed.

Many height-dbh models have been developed and used to estimate tree height from dbh [12]. A large number of generalised height-dbh equations have been reported that have been developed especially for a particular species or for specific areas. The relationship between height and dbh of even-aged stands can be expressed by linear functions, such as second-order polynomial equations. Curtis [13] summarised a large number of available height-dbh models and used the Furnival Index to evaluate the performance of linear functions fitted to second-growth Douglas fir data. The commonly used functions are, however, nonlinear. Huang et al. [14] evaluated 20 nonlinear height-dbh models for major Alberta species. Ecoregion-based height-dbh models have also been developed [5, 15–17]. With the relative ease of fitting nonlinear functions, the nature of nonlinear height-dbh functions has now been widely used in height predictions [18–21]; however, an expansion of the predictions would probably lead to biased predictions, owing to variability of the height-dbh relationship. This relationship is highly dependent upon the growth conditions and upon stand characteristics such as stand density, stand age, basal area, site index, mean and dominant heights and diameters. The height-dbh relationship was variable across different stands and even changed with time in the same stand. Therefore, we could not use a simple function for all possible relationships between height and dbh. Mixed-model methods have been used to estimate fixed and random-effect parameters for some height-dbh functions. The random effects specific to each plot allowed for the lack of independence among observations derived from the special hierarchical structure of the data (trees within plots). Moreover, Castedo et al. [22] pointed out that the mixed-effects model provided better model fitness and more precise estimates than the corresponding basic generalised model. As for mixed-effects models, local height-dbh models adequately described the relationship between both tree characteristics at the stand level, if derived from a sufficiently representative sample of diameter-height measurements, and were often used in forest inventories [7]. However, there have been very few in-depth studies on height-dbh relations for Chinese fir plantations.

Chinese fir (*Cunninghamia lanceolata* (Lamb.) Hook) is a principal native tree species in subtropical areas of southern China and is used extensively as the main commercial species for construction, railroad ties, mine timber, furniture, wood pulp and other purposes. There is still a knowledge gap regarding the height-dbh relationship for Chinese-fir plantations. Therefore, the aim of this study were to: 1)develop a model that could be used to predict the height-dbh relationship for Chinese-fir plantations in central southern China; 2) determine which stand variables influenced the height-dbh relationship significantly.

## Materials and Methods

### Ethics Statement

Our study sites (Dashanchong Forest Farm and Huitong) are owned by the Forestry Bureau of Changsha county and Huitong county, Hunan Province and managed by the Dashanchong Forest Farm and Forestry Bureau of Huitong, respectively. We conducted our research works by the permission in the collaboration contract with Dashanchong Forest Farm and Forestry Bureau of Huitong, and under the Regulations of the People’s Republic of China on State Forest Farm. Our field studies did not involve endangered or protected species.

### Site description

**Fig 1:**
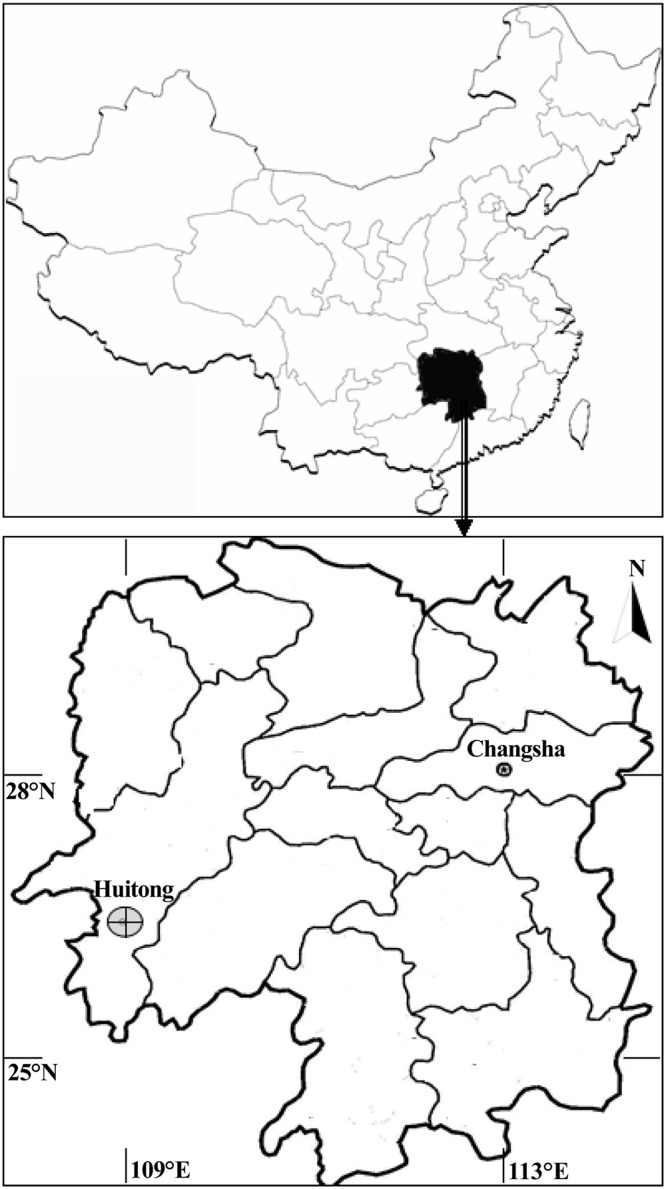
Geographical locations of the study sites.

The study site were located in Huitong and Changsha counties, Hunan Province, China (Lat. 24°38′ to 30°08′ N and Long. 108°47′ to 114°15′ E) (Fig 1). The study area is in a humid, subtropical monsoon region with annual precipitation of 1,400 mm, mean annual average temperature of 16.8°C and mean annual average evapotranspiration of 1322 mm, and the frost-free period is 278–300 d. Elevation of the study sites is 280–390 m and 55–220 m in Huitong and Changsha, respectively. The soil of the study site in Huitong is a red, clay loam soil originating from shale and slate parent rocks, and in Changsha it is a red soil on a slate parent rock. In order to include a range of stand conditions and silvicultures, the sample trees were selected from plots of different characteristics within subtropical plantations in these regions. A total of 80 Chinese-fir plantation plots, ranging in age from 6 to 53 years were selected within the study areas. Each plot was of 666.7 m^2^ (fixed size) and square in shape with a side of 25.82 m. The maximum age, dbh and height of mature trees in these plots were 53 a, 43.9 cm, and 27.9 m, respectively. And the average age, dbh and height of mature trees in these plots were 48 a, 26.8 cm, and 19.1 m, respectively.

### Data collection

In all plots, the dbh (d in cm) of all trees larger than 2 cm was measured. The following stand-level variables were calculated for each plot, based on data from a complete survey: stand density (N in stems·ha^-1^), basal area (BA in m^2^·ha^-1^), quadratic mean dbh (Dq in cm). The five highest dominant trees and about 50 sample trees were selected randomly for each plot and, for each tree, the dbh, the height (h in m), the height below the lowest live branch and the crown width were measured. The stand mean height (Hm in m) was calculated using the accumulated basal area data from the sample trees. Other stand-level variables, such as the dominant dbh (D0 in cm) and the dominant height (H0 in m), were calculated from the accumulated basal area data from the five highest dominant trees in each plot. In total, there were data from 5961 Chinese-fir tree samples available for this study.

The data for the 5,961 sample trees were randomly divided into two parts. The majority of the data (80%) were used for model calibration, and the remainder (20%) were used for model validation. For the calibration data, the mean dbh and height were 13.0 cm and 10.70 m, respectively, whereas for the validation data, the mean dbh and height were 13.9 cm and 10.87 m, respectively. Descriptive stand characteristics, including stand-level variables, are provided in Table 1.

**Table 1:** Descriptive statistics of the sample trees in the plots of Chinese-fir.

	Variable	Mean	Maximum	Minimum	Standard deviation
Calibration data (n = 4769)	h	10.70	27.90	1.40	4.30
	d	13.0	43.9	2.0	6.20
	BA	25.92	47.31	9.07	8.97
	Dq	13.44	32.61	7.79	5.09
	D0	19.64	38.22	12.62	5.39
	Hm	11.81	23.20	5.71	3.47
	H0	15.29	26.65	8.03	3.72
	N	1999	2715	400	482.86
	t	15	53	6	11.34
Validation data (n = 1192)	h	10.87	26.70	2.00	4.26
	d	13.9	42.0	2.0	6.22
	BA	26.06	47.30	9.07	8.78
	Dq	13.46	32.61	7.79	5.02
	D0	19.67	38.22	12.62	5.30
	Hm	11.84	23.20	5.71	3.39
	H0	15.35	26.65	8.03	3.65
	N	2002	2715	400	483.99
	t	15	53	6	11.33

h: height, m; d: diameter at breast height (dbh), cm; BA: basal area, m^2^·ha^-1^; Dq: quadratic mean dbh of the stand, cm; D0: dominant dbh of the stand, cm; Hm: mean height of the stand, cm; H0: dominant height of the stand, m; N: number of trees per hectare; t: age of the stand.

### Model description and selection criteria

Both linear and nonlinear models were used here in order to compare their performance. In this study, we considered the most commonly used models, especially developed for Chinese-fir. Finally, we analysed two groups of generalised height-dbh models given in Table 2, making 39 equations in total. Based on the number of independent variables, two groups were established, as follows:

**Table 2:** Height-dbh models selected for comparison.

No.	Model	References	Group
Liner models			
1	*h* = *a*_0_+ *a*_1_ *d*	[2]	1
2	*h* ^-1^ = *a*_0_+ *a*_1_ *d* ^-1^	[35]	1
3	log(*h*-1.3) = *a*_0_+ *a*_1_ log *d*	[13, 36]	1
4	*h = a*_0_+ *a*_1_ *d+ a*_2_ *d* ^2^	[13, 37]	1
5	*h* = *a*_0_ + *a*_1_(*d* / *D*_*q*_)+ *a*_2_ *H*_*m*_	[2]	2
6	log(*h*-1.3) = *a*_*0*_ *+ a*_1_ log(*d/D*_*q*_) + *a*_2_ log(*H*_*m*_)	[2]	2
Nonlinear models			
7	$h=1.3+a_{0}d^{a_{1}}$	[38–39]	1
8	$h=1.3+e^{a_{0+}a_{1}/(d+1)}$	[18]	1
9	*h* = 1.3 + *a*_0_ *d/*(*a*_1_ + *d*)	[20]	1
10	$h=1.3+a_{0}(1-e^{-a_{1}d})$	[40]	1
11	$h=1.3+10^{a_{0}}d^{a_{1}}$	[41–42]	1
12	*h* = 1.3 + *a*_0_ *d/*(*d*+1)+ *a*_1_ *d*	[43–44]	1
13	$h=1.3+a_{0}{\left(d/(1+d)\right)}^{a_{1}}$	[13, 45]	1
14	$h=1.3+a_{0}/(1+a_{1}e^{-a_{2}d})$	[46]	1
15	$h=1.3+a_{0}{\left(1-e^{-a_{1}d}\right)}^{a_{2}}$	[47]	1
16	$h=1.3+a_{0}(1-e^{-a_{1}d^{a_{2}}})$	[48–49]	1
17	$h=1.3+a_{0}e^{-a_{1}e^{-a_{2}d}}$	[50]	1
18	*h* = 1.3 + *d* ^*2*^/(*a*_0_+ *a*_1_ *d*+ *a*_2_ *d* ^2^)	[13, 45]	1
19	$h=1.3+a_{0}d^{a_{1}d^{-a_{2}}}$	[51]	1
20	$h=1.3+a_{0}{}^{e^{a_{1/(d+a_{2})}}}$	[52]	1
21	$h=1.3+a_{0}/(1+a_{1}{}^{-1}d^{-a_{2}}{}_{})$	[53]	1
22	*h* = 1.3 + *a*_0_ *+a*_1_/(*d* + *a*_2_)	[54]	1
23	$h=1.3+a_{0}e^{\left(-a_{1}d^{-a_{2}}\right)}$	[55–56]	1
24	h=1.3+a0[e−e(−a(d−a2)1)]	[57]	1
25	$h=1.3+e^{\left(a_{0+}a_{1}d^{e_{2}}\right)}$	[19, 58–59]	1
26	$h=1.3+e^{\left(a_{0}+a_{1}d^{e_{2}}\right)}$	[13]	1
27	$h=1.3+a_{0}{\left(BA\right)}^{a_{1}}(1-e^{-a_{2}d})$	[24]	2
28	$h=1.3+(H_{m}-1.3)e^{a_{0}(1-\frac{d}{Dq})+a_{1}(\frac{d}{Dq}-\frac{1}{d})}$	[23, 60]	2
29	$h=H_{0}(1+a_{0}e^{a_{1}H_{0}})(1-e^{\frac{-a_{2}d}{H_{0}}})$	[61]	2
30	$h=1.3+a_{0}H_{0}{}^{a_{1}}d^{a_{2}H_{0}^{a_{3}}}$	[62]	2
31	$h=1.3+({\text{a}}_{0}+a_{1}H_{0}-a_{2}D_{q})e^{-a_{3}/d}$	[23, 63]	2
32	$h=1.3+({\text{a}}_{0}+a_{1}H_{0}-a_{2}D_{q})e^{-a_{3}/\sqrt{\text{d}}}$	[23]	2
33	$h=1.3+({\text{a}}_{0}+a_{1}H_{0}-a_{2}D_{q}+a_{3}BA)e^{-a_{4}/\sqrt{\text{d}}}$	[23]	2
34	$h=a_{0}+a_{1}H_{m}+a_{2}D_{q}^{0.95}+a_{3}e^{-0.08d}+a_{4}H_{m}^{3}e^{-0.08d}+a_{5}D_{q}^{3}e^{-0.08d}$	[23, 64]	2
35	$h=10^{\left(a_{0}+a_{1}\frac{1}{d}+a_{2}\frac{1}{t}+a_{3}\frac{1}{D_{q}t}\right)}$	[65]	2
36	$h=e^{\left(a_{0}+a_{1}\ln D_{q}+a_{2}\ln N+a_{3}\sqrt{d}\right)}$	[64]	2
37	$h=a_{0}+a_{1}H_{m}+a_{2}D_{q}+a_{3}e^{a_{4}d}+a_{5}H_{m}^{a_{6}}e^{a_{4}d}+a_{7}D_{q}^{a_{8}}e^{a_{4}d}$	[23, 64]	2
38	$h=e^{a_{0}+a_{1}\ln H_{0}+a_{2}\frac{1}{t}+a_{3}\frac{\ln N}{d}+a_{4}\frac{1}{dt}+a_{5}\frac{1}{d}}$	[42]	2
39	$h=a_{0}H_{0}^{a_{1}}{\left(BA\right)}^{a_{2}}N^{a_{3}}e^{\frac{a_{4}}{t}+\frac{a_{5}}{d}}$	[66]	2

h: height, m; d: diameter at breast height (dbh), cm; BA: basal area, m^2^·ha^-1^; Dq: quadratic mean dbh of the stand, cm; D0: dominant dbh of the stand, cm; Hm: mean height of the stand, cm; H0: dominant height of the stand, m; N: number of trees per hectare; t: age of the stand; a0-a8 are parameters. The base is 10 for logarithm.

Group 1: models using one independent variable, requiring only dbh measurements.

Group 2: models using two or more independent variables, requiring measurements of dbh and other stand characteristics.

Model evaluation and comparison were based on graphical and numerical analysis of the values of the following statistics: 1) root of mean square error (RMSE) (Eq 1), which analysed the precision of the estimates (the smaller, the better); 2) adjusted coefficient of determination (R^2^adj) (Eqs 2 and 3), which reflected the part of the total variance explained by the model and which took into account the number of parameters that were necessary in making the estimates (the greater the value, the higher the interrelation between the variables); 3) bias (Eq 4) and relative bias (Eq 5), which evaluated the deviation of the model with respect to the observed results (the smaller, the better); and 4) Akaike’s information criterion (AIC) (Eq 6 and Eq 7), which is a commonly used information criterion and which was used to select the best model (as a rule, the model with the lower AIC values was preferred). The expressions for these statistics are as follows:

$$\text{Root of mean square error}:RMSE=\sqrt{\frac{\sum_{i=1}^{n}{\left(y_{i}-\hat{y_{i}}\right)}^{2}}{n-p-1}}$$ (1)

$$\text{Coefficient of determination: }R^{2}\;=\;1-\frac{\sum_{i=1}^{n}{\left(y_{i}-{\hat{y}}_{i}\right)}^{2}}{\sum_{i=1}^{n}{\left(y_{i}-{\overset{-}{y}}_{i}\right)}^{2}}$$ (2)

$$\text{Adjusted coefficient of determination: }R_{adj}^{2}\;=\;1-(1-R^{2})•\frac{n-1}{n-p-1}$$ (3)

$$\text{Bias }=\;\frac{\sum_{i=1}^{n}\left({\hat{y}}_{i}-y\right)}{n}$$ (4)

$$\text{Relative bias }\left(\text{\%}\right)\;=\;100•\frac{\sum_{i=1}^{n}({\hat{y}}_{i}-y)/y_{i}}{n}$$ (5)

$$\text{Residual sum of squares:}RSS\;=\;\sum_{i=1}^{n}{\left(y_{i}-{\hat{y}}_{i}\right)}^{2}$$ (6)

Akaike’s information criterion:AIC = nln(RSS)+2(p+1)−nln(n) (7)

where $y_{i},\;{\hat{y}}_{i}\;and\;\bar{y}$ are the observed, predicted and mean values of heights, respectively; n is the total number of data used in fitting the model; and p is the number of independent variables.

### Data analysis

Most of the models described above are nonlinear, so model fitting was carried out using the SPSS statistical program package 13.0 (SPSS for Windows, Version 13.0, SPSS, Chicago, IL, USA) and the Levenberg-Marquardt (LM) method. The initial values of the parameters for starting the iterative procedure were obtained, where possible, by previously linearising the equation and fitting it to the data by ordinary least squares, using the regression procedure of the SPSS programme.

## Results

### Variation in h-dbh relationship among variables

**Fig 2:**
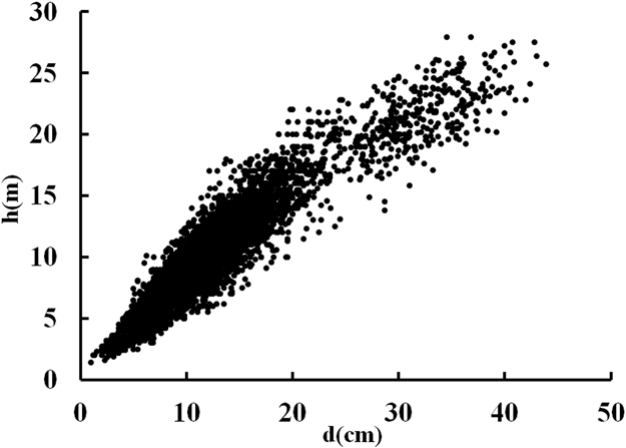
Scatter graph of individual height and dbh in Chinese-fir plantations.

**Fig 3:**
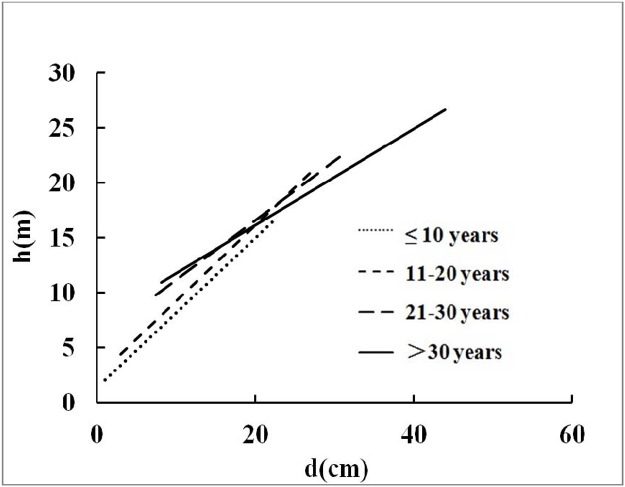
The linear trend line of height-dbh for different ages.

**Fig 4:**
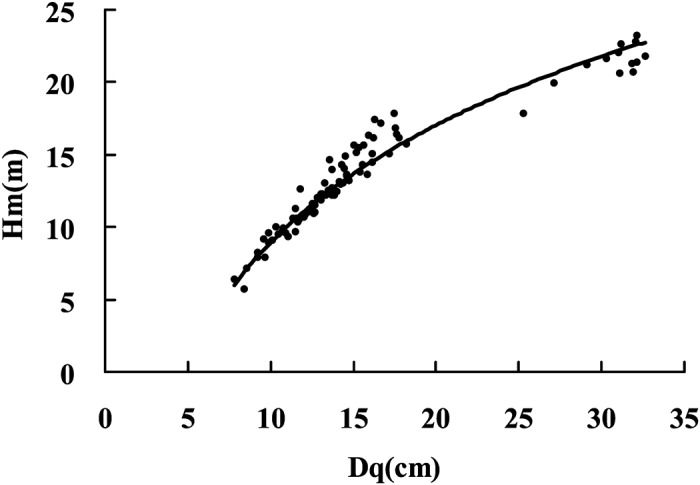
The scatter plot and logarithmic trend line for stand Dq and Hm. The solid line represents the trend line.

The scatter plot of the individual height and dbh values for individual trees of Chinese-fir plantations is presented in Fig 2. At dbh values less than 20 cm, tree height increased rapidly as dbh increased; however, as the dbh increased further, the increase in tree height slowed down and the height-dbh curve became less steep. Fig 3 shows the trend lines for the height-dbh relationship for stands of different ages; the slope of the trend line varied according to stand age. Fig 4 shows the scatter plot and the logarithmic trend line for stand Dq and Hm values. The solid line depicts the trend line. The function of the trend line was Hm = 11.707×ln(Dq)-18.032 (R^2^ = 0.9577). It was therefore possible to predict Hm from Dq. Consequently, from a predictive point of view, in influencing the height-dbh relationship, Dq was revealed to be the most important stand variable.

### Model fitting and selection

Results of goodness of fit and prediction accuracy for the calibration and validation datasets are given in Table 3 for the Group 1 models and in Table 4 for the Group 2 models. The adjusted R^2^ and RMSE values were approximately equivalent among the Group 1 models (Table 3). Among the Group 1 models, Model 4 produced the best fit to the data (the highest adjusted R^2^ and the lowest RMSE, bias and AIC) in the calibration dataset and could be considered as the equation with most accuracy for tree height estimation in Group 1. This equation only used one independent variable (dbh) for predicting the height and produced a good fit to the data. When stand variables were taken into account (i.e. Group 2 models), different performances were revealed amongst the models. For the Group 2 models, the adjusted R^2^ values ranged from 0.8667 to 0.9136 and the RMSE values ranged from 1.2627 to 1.5827 in calibration dataset (Table 4). Furthermore, most Group 2 models had a larger adjusted R^2^ and a smaller RMSE than the Group 1 models. The values for AIC in the calibration dataset were very high among the Group 1 models. The value of AIC decreased by 2,158.1 in the calibration dataset for Group 2 models. For selection, models were ranked in terms of their performance (adjusted R^2^, RMSE, absolute bias, relative bias and AIC) for the calibration and validation datasets. In this analysis, models that ranked within the first two or three are summarised in Table 5 (with a number in parenthesis indicating the rank of the model according to the respective attribute). With respect to adjusted R^2^, the model with the value closest to one was the highest-ranking, whereas for bias (both absolute and relative bias), the model with the value closest to zero was considered to be the best. For RMSE and AIC, the model with the lowest value had the highest ranking. For each model, its ranking for the five evaluation statistics was summated. The model with the smallest sum total (i.e. the highest overall ranking) was considered to be the best growth function for each of the Chinese-fir databases. According to this analysis, amongst the Group 1 models, Model 4 was the best model for the calibration dataset, whereas Model 16 was the best for the validation dataset. Model 15 ranked similarly for both the calibration and validation datasets. On the whole, there was little difference amongst the Group 1 models. The best-performing models—not only in Group 2 but across all models—were the nonlinear models, Model 37 and Model 34, followed by the linear model, Model 6. The nonlinear models therefore performed a little better than the linear models.

**Table 3:** Fitting statistics for Group 1 models using both calibration and validation datasets.

Model	Variables	Calibration				Validation		
		RMSE (m)	Adjusted R^2^	Bias (m)	AIC	RMSE (m)	Bias (m)	AIC
1	h,d	1.6472	0.8530	0.0000	4764.3	1.6656	0.0000	1220.3
2	h,d	1.5352	0.8725	0.0150	4092.4	1.5297	0.0228	1017.4
3	h,d	1.5989	0.8624	0.0467	4480.3	1.6083	0.0501	1136.8
4	h,d	1.5299	0.8732	0.0000	4059.6	1.5163	0.0000	996.4
7	h,d	1.5989	0.8624	0.0467	4480.3	1.6083	0.0501	1136.8
8	h,d	1.6132	0.8607	-0.0711	4565.4	1.5859	-0.0611	1103.3
9	h,d	1.5650	0.8687	0.0585	4276.0	1.5655	0.0646	1072.5
10	h,d	1.5625	0.8692	0.0605	4260.9	1.5618	0.0675	1066.9
11	h,d	1.5989	0.8624	0.0467	4480.3	1.6083	0.0501	1136.8
12	h,d	1.5650	0.8687	0.0585	4276.0	1.5655	0.0646	1072.5
13	h,d	1.6375	0.8571	-0.0844	4708.1	1.6077	-0.0729	1136.0
14	h,d	1.5782	0.8653	0.0226	4356.3	1.5535	0.0196	1054.2
15	h,d	1.5303	0.8732	0.0020	4061.9	1.5166	0.0001	996.8
16	h,d	1.5305	0.8731	0.0034	4063.3	1.5161	0.0010	996.1
17	h,d	1.5449	0.8708	0.0096	4152.5	1.5255	0.0077	1010.9
18	h,d	1.5308	0.8731	-0.0008	4065.1	1.5192	-0.0026	1000.9
19	h,d	1.5313	0.8730	-0.0018	4068.4	1.5203	-0.0035	1002.6
20	h,d	1.5329	0.8727	0.0040	4078.6	1.5194	0.0028	1001.3
21	h,d	1.5304	0.8732	0.0015	4062.7	1.5174	-0.0005	998.1
22	h,d	1.5324	0.8728	0.0000	4075.3	1.5229	0.0000	1006.8
23	h,d	1.5319	0.8729	-0.0020	4071.8	1.5216	-0.0034	1004.6
24	h,d	1.5449	0.8708	0.0096	4152.5	1.5255	0.0077	1010.9
25	h,d	1.5319	0.8729	-0.0020	4071.8	1.5216	-0.0034	1004.6
26	h,d	1.5319	0.8729	-0.0020	4071.8	1.5216	-0.0034	1004.6

**Table 4:** Fitting statistics for Group 2 models using both calibration and validation datasets.

Model	Variables	Calibration				Validation		
		RMSE (m)	Adjusted R^2^	Bias (m)	AIC	RMSE(m)	Bias (m)	AIC
5	h,d,Dq,Hm	1.3881	0.8956	0.0000	3136.0	1.3622	0.0000	745.0
6	h,d,Dq,Hm	1.2994	0.9088	0.0256	2505.7	1.3316	0.0313	690.6
27	h,d,BA	1.5277	0.8736	0.0004	4048.0	1.5204	0.0177	1004.9
28	h,d,Dq,Hm	1.4634	0.8902	0.1397	3639.6	1.4513	0.1264	896.0
29	h,d,H0	1.4480	0.8873	0.0437	3537.1	1.4541	0.0464	898.5
30	h,d,H0	1.4729	0.8830	0.0372	3699.3	1.4667	0.0388	919.1
31	h,d,Dq,H0	1.4479	0.8866	-0.0254	3538.0	1.4252	-0.0211	852.8
32	h,d,Dq,H0	1.4270	0.8897	0.0051	3399.5	1.4099	0.0066	826.9
33	h,d,Dq,H0,BA	1.3911	0.8951	0.0041	3158.3	1.3861	0.0051	788.4
34	h,d,Dq,Hm	1.2757	0.9118	0.0000	2330.1	1.3081	0.0000	648.3
35	h,d,Dq,t	1.4381	0.8890	-0.0505	3473.3	1.4216	-0.0439	846.6
36	h,d,Dq,N	1.5657	0.8682	0.0468	4284.2	1.6028	0.0468	1132.6
37	h,d,Dq,Hm	1.2627	0.9136	0.0026	2233.0	1.2739	0.0000	585.2
38	h,d,H0,t,N	1.3944	0.8948	-0.0224	3181.3	1.3887	-0.0206	792.8
39	h,d,H0,BA,N,t	1.5827	0.8667	-0.0788	4391.1	1.5357	-0.0631	1034.7

**Table 5:** Model rank based on performance.

Model performance	Group 1 models		Group 2 models		All models	
	Calibration data	Validation data	Calibration data	Validation data	Calibration data	Validation data
Adjusted R^2^	4-15-21(1); 16-18(2)	4-16(1); 15(2); 21(3)	37(1); 34(2); 6(3)	37(1); 34(2); 6(3)	37(1); 34(2); 6(3)	37(1); 34(2); 6(3)
RMSE	4(1); 15(2); 21(3)	16(1); 4(2); 15(3)	37(1); 34(2); 6(3)	37(1); 34(2); 6(3)	37(1); 34(2); 6(3)	37(1); 34(2); 6(3)
AIC	4(1); 15(2); 21(3)	16(1); 4(2); 15(3)	37(1); 34(2); 6(3)	37(1); 34(2); 6(3)	37(1); 34(2); 6(3)	37(1); 34(2); 6(3)
Absolute bias	1-4-22(1); 18(2)	22-4-1(1); 15(2)	5-34(1); 27(2); 37(3)	37-34-5(1); 33(2)	34-22(1); 5-4(2)	5-34-37-22-4-1(1); 15(2)
Relative bias	13(1); 22(2); 25(3)	13(1); 20(2); 8(3)	39(1); 35(2); 38(3)	35(1); 39(2); 38(3)	39(1); 13(2); 35(3)	13(1); 35(2); 39(3)

Numbers in parenthesis indicate the rank of the model for the attributes in the table. Rank one represents the best performance.

**Fig 5:**
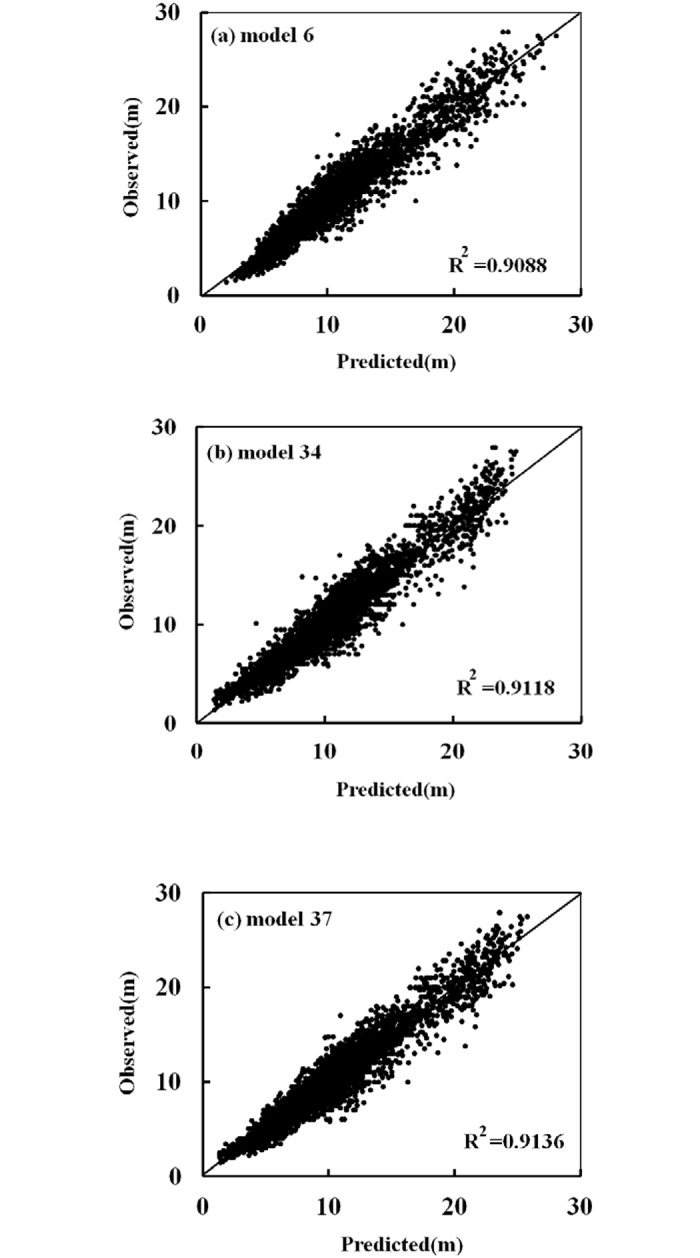
Graph of observed values versus predicted values in the calibration dataset for the three best models (a: Model 6; b: Model 34; c: Model 37). The solid line represents the diagonal.

**Fig 6:**
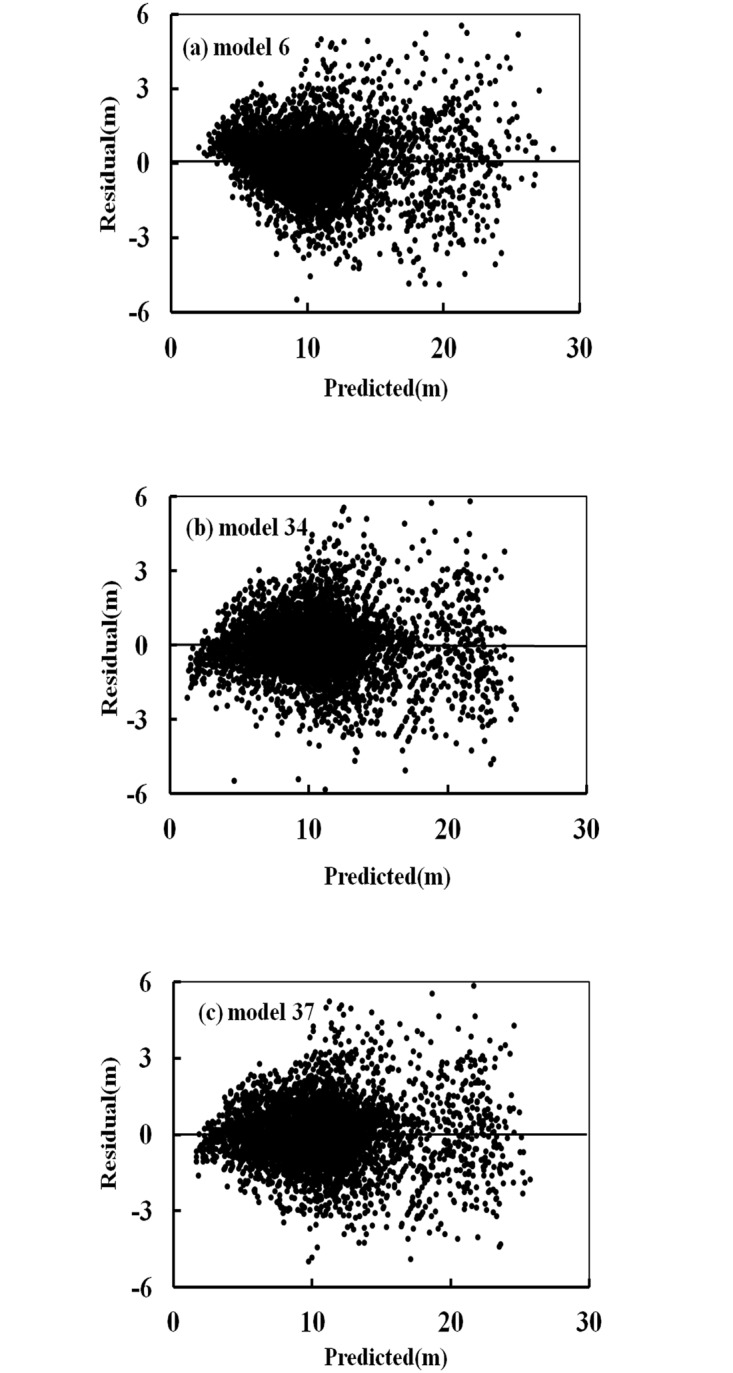
Residual plots in the calibration dataset for the three best models (a: Model 6; b: Model 34; c: Model 37).

**Fig 7:**
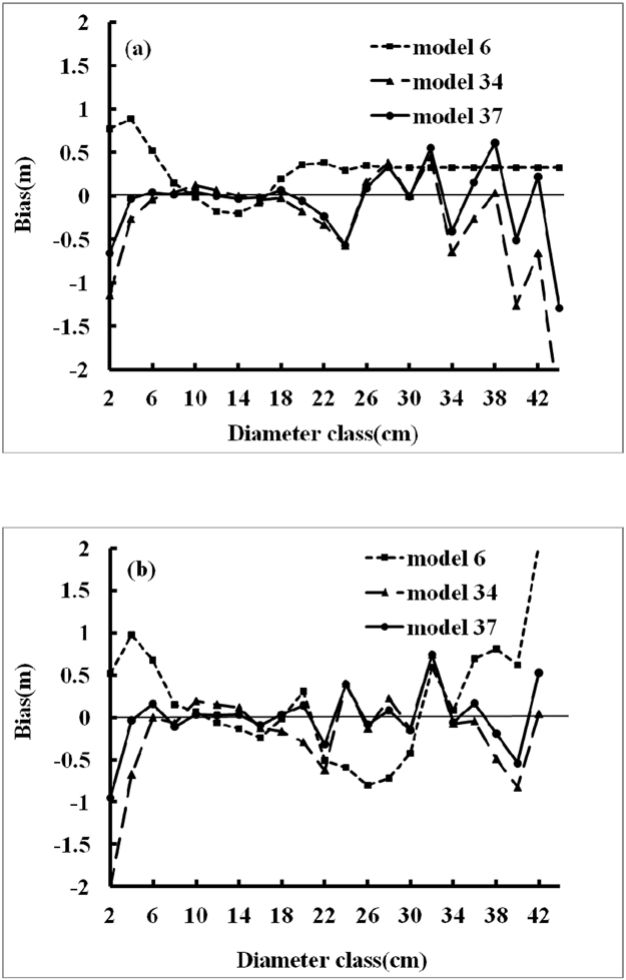
Values of average bias in relation to dbh in the calibration and validation datasets for the two best models (a: calibration data; b: validation data).

The observed heights versus the predicted heights for these models, for all datasets, are shown in Fig 5. The criterion used to evaluate the performance of a model was the determination coefficient of the straight line between the observed and predicted heights (i.e., the solid line represents the diagonal). Each model had a relatively high R^2^, so the solid line was closely surrounded by the data points. No significant tendency towards overestimation or underestimation of height values was observed, except for the overestimation in Model 6 (Fig 5a) when tree height was small. The residuals versus the predicted height in the calibration dataset of Models 37, 34 and 6 are shown in Fig 6. Most data points were distributed around the zero line. These models showed an approximately homogeneous variance over the full range of the predicted values, as well as independence of the residuals. Fig 7 shows the values for the average bias obtained with respect to diameter, in the calibration and validation datasets for Models 6, 34 and 37. The biases for large and small values of diameter were greater than the biases for intermediate values.

From the analysis described above, Models 37, 34 and 6 emerged as the three best models in this study. The number of parameters used in Model 37, however, was quite high. For this reason, Models 34 and 6, which used a smaller number of parameters (6 and 3 parameters, respectively), were selected as the final models in this study.

### Parameter estimates

**Fig 8:**
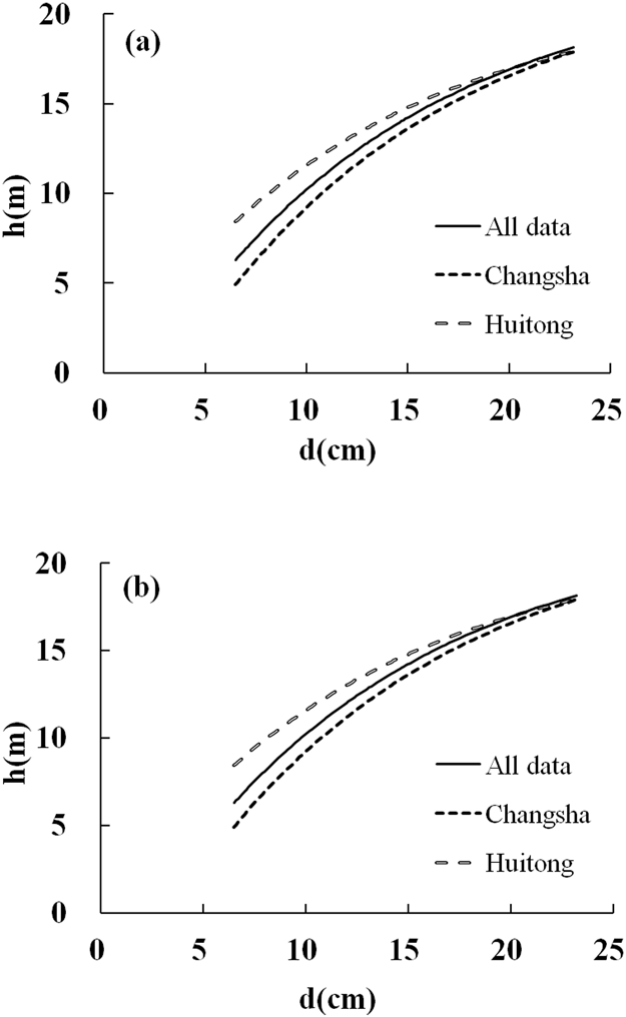
The height-dbh relationship for the two best models, using the following data: Dq = 15.04cm, Hm = 15.64m, D0 = 22.52cm, H0 = 22.51m, t = 18 years old, N = 2302 trees per hectare (a: Model 6; b: Model 34).

Initial development of the models was conducted using only 80% of the dataset and the parameters for Chinese-fir were estimated for all of the models. For Models 34 and 6, the parameter estimates and fitting statistics were calculated using all of the dataset and are shown in Table 6. All parameters were significant (p < 0.05). The parameters for each model were easily obtained using the calculation procedures. Table 7 shows the parameter estimates for the final two models for the various different age classes. The parameter estimates also varied between the two sites (Table 8). Fig 8 shows the relationship line for the final height-dbh models across different sites. We used fixed Dq (15.04 cm) and Hm (15.64 m) values for the final models within specific diameter ranges (6.5 to 23.2 cm). It was clear that the fitting of the models for all datasets was overestimated for Changsha and underestimated for Huitong.

**Table 6:** Parameter estimates and fitting statistics of the final models using all data.

Parameter	Model 34		Model 6	
	Estimate value	Standard error	Estimate value	Standard error
a0	8.0870	0.2097	-0.2728	0.0063
a1	1.1628	0.0422	0.8009	0.0069
a2	-0.2973	0.0320	1.1728	0.0054
a3	-14.3844	0.2393	-	-
a4	-0.0033	0.0002	-	-
a5	-3.5353	0.0001	-	-
Adjusted R^2^	0.9105		0.9075	
RMSE (m)	1.2830		1.3060	
Absolute bias (m)	0.0000		0.0000	
Relative bias (%)	1.1401		1.5472	

**Table 7:** Parameter estimates of the final models in different age classes.

Model	Age class	Parameter					
		a0	a1	a2	a3	a4	a5
Model 6	≤10	-0.4152	0.8866	1.3156	-	-	-
	11–20	-0.1204	0.8433	1.0335	-	-	-
	21–30	-0.0933	0.6036	1.0218	-	-	-
	>30	-0.3425	0.6332	1.2284	-	-	-
Model 34	≤10	6.3448	1.2792	-0.2393	-11.9213	-0.0054	-0.0004
	11–20	6.3448	1.2104	-0.1579	-12.8419	-0.0022	-0.0021
	21–30	8.1797	0.7992	0.0632	-15.8155	-0.0014	-0.0007
	>30	2.4538	1.5721	-0.4220	-6.1383	-0.0045	0.0002

**Table 8:** Parameter estimates of the two best models in different regions.

Model	Region	Parameter					
		a0	a1	a2	a3	a4	a5
Model 6	Changsha	-0.2876	0.6486	1.1873	-	-	-
	Huitong	-0.1616	0.5937	1.0820	-	-	-
Model 34	Changsha	5.2595	1.6454	-0.5949	-16.0497	-0.0058	0.0010
	Huitong	7.3952	1.0349	-0.1726	-14.3900	-0.0014	-0.0015

## Discussion

### Relationship between height and stand variables

Previous studies had shown that the inclusion of stand characteristics as independent variables in height-dbh models improved the prediction accuracy of tree height estimation [23–26]. Stand variables reported in the literature include dominant dbh, dominant height, stand age, number of trees per hectare, stand basal area, density stress, developmental status and the combination of density stress and developmental status. This conclusion has been reconfirmed for Chinese-fir plantations in this study, in which height-dbh models for Chinese-fir have been assessed for practical application in subtropical China. Group 1 models can be used to predict the height-dbh relationship for a stand using only dbh (d) as independent variable. If other stand characteristics are measured—such as mean height (Hm), quadratic mean dbh (Dq), dominant dbh of the stand (D0), mean height of the stand (Hm), dominant height of the stand (H0), number of trees per hectare (N) and age of the stand (t)—then a choice can be made from Group 2 models, based on the best model for the particular situation.

Measured height-dbh pairs can be used in refining the model for use with a particular target stand, and information regarding development status can be used (i.e. the specific stand age). Furthermore, the future height-dbh curve for a stand can be predicted, though this requires the prediction of Hm and Dq. In this study, the models containing Dq and Hm predicted tree height very effectively. Dq could be calculated easily from the diameters at breast height of all trees in the stands, but Hm required measurements of sample trees to be taken; any bias in the selection of sample trees would prejudice the accuracy of the resulting height prediction. The scatter plot and logarithmic trend line for stand Dq and Hm values are shown in Fig 4, and it is clear that Hm could be predicted easily from Dq. Thus, from a predictive viewpoint, Dq was the most important stand variable to influence the height-dbh relationship.

The variation in the height-dbh relationship for stands of different ages is shown in Fig 3. Owing to the allometric relationship between tree height and dbh, the rate of increase of tree height was significantly different from that of dbh. Though the stand age was not the most important variable in the tree height-dbh relationship, some differences in parameter estimates were found among the selected models. Therefore, tree age should be considered when choosing models and parameters aimed at predicting tree height more accurately. Sánchez [23] found that the inclusion of stand age and density contributed to model performance for even-aged *Pinus radiata* stands. Vanclay [27] also reported that stand density played an important role in even-aged pure plantations. However, Mehtätalo [28] observed that the development of the height-dbh curve for a shade-tolerant tree species, Norway spruce, depended upon the stand average tree size rather than upon stand age. This study presented a similar result, in that the stand age (t) and density (N) in the models were less significant than Hm and Dq; this is explicable because Dq and Hm reflect variations in stand age and density. Furthermore, the planting density of Chinese-fir plantation in this study was initially identical. With increasing stand age, stand density would be decreased by intermediate cutting, and this would create a different stand density. Given sufficient growth space, the different stand density would not affect tree growth. Site properties also affect the rate of development of forest stands. That is why forest stands on poor sites develop more slowly and take longer to reach maturity, compared to stands on fertile sites.

Given that tree height and dbh usually increased with tree age and were influenced by density, the models predicting the development of the height-dbh curve for a stand performed better when Hm and Dq were used as the variables describing the stand rather than stand age and density. This effect on the performance of the models was probably true for aspects of model prediction other than the height-dbh curves. Hence, when modelling the development of any stand characteristics, e.g. diameter distribution and stand growth, the use of stand age as the sole variable for determining the stage of development of the stand should be viewed critically.

The relationship line for the final height-dbh models at different sites (Fig 8) showed that the fitting of the models for all datasets was overestimated for Changsha and underestimated for Huitong. The results could be attributed to the differences in local climate and soil conditions between the two sites. Comparing Chinese-fir plantations grown at different sites, the yields at sites in the central Chinese-fir producing area were usually higher than at sites in hilly areas. The mean annual temperature in Huitong is 16.5°C, which ranges from an average of 4.3°C during the coldest month (January) to 27.1°C [29] during the warmest month (July) [29–31]; while the mean temperature is 17.1°C for Changsha with mean temperature of 4.9°C in January and 29.0°C in July [29,31]. It was reported that the difference in meteorological factors between Huitong and Zhuting (adjacent to Changsha, about 50 km away) indicated that the climate in Huitong was more favourite for the growth of Chinese-fir plantation, with stand production of 9.18 ×10^4^ kg·hm^-2^ for Huitong (mean dbh of 11.7 cm) and 5.93×10^4^ kg·hm^-2^ for Zhuting (mean dbh of 8.2 cm) both at stand age of 11a [31]. Huitong is the main production area for Chinese-fir in the central southern part of China and the soil conditions there are especially suitable for the growth of Chinese-fir. Our results were consistent with the study of Pan et al. [31]. As a consequence, the parameter estimates therefore also varied between the two sites (Table 8).

### Model evaluation and application

Model selection was based on goodness of fit, precision and practical application. Model comparisons were carried out based on the ranking (Table 5). Using this approach, the Group 1 models could not be clearly differentiated. This result may be explained by the monotonic increment of tree height with dbh, as shown in Fig 2. It may coincide with the property of the first monotonic increment of a reasonable height-dbh model format, as suggested by Lei and Parresol [32], which should possess the S-shaped functional properties of monotonic increment, inflection point and asymptotical value. In the case of the Group 2 models, Models 37, 34 and 6 were the most consistent between calibration and validation data. In general, the inclusion of new independent variables in the height-dbh model reduced bias and increased the precision of the model. So, with a larger sampling effort and a greater number of stand variables measured in the field, the Group 2 models performed better than the Group 1 models in tree height prediction.

Specifically, Models 34 and 6 provided a relatively accurate prediction for this tree species using dbh (d), mean height (Hm) and quadratic mean diameter (Dq) as independent variables. The positive performance of Model 6 may be due, in part, to the inclusion of the competition index (d/Dq), represented as the ratio of individual tree diameter and quadratic mean diameter, an important variable for the social status of trees [33], especially for plantations under competition stress. Vargas-Larreta [34] also found that the model which included the dbh (d) and the quadratic mean diameter (Dq) and the dominant height of the stand (H0) as independent variables predicted well in uneven-aged stands. Therefore, Models 34 and 6, which used dbh (d), mean height (Hm) and quadratic mean diameter (Dq) as independent variables, displayed the best performance and showed consistency between the calibration and validation data, and were therefore selected as the final models.

Lei et al. [2], however, showed that a linear mixed-effect model performed better than nonlinear mixed-effect models for young black spruce and jack pine plantations. Tree height and diameter relationships are generally described using nonlinear models. In the present study, we found that nonlinear mixed-effect models performed better than linear mixed-effect models for Chinese-fir plantations. This may be a consequence of the rapid growth of Chinese-fir and the warm and humid climate in this particular area. Furthermore, nonlinear models are more flexible than linear models.

From the analysis above, Models 37, 34 and 6 were the best three models in this study. There were some similarities between Models 37 and 34 and the fitting statistics of the two models were almost equal. The number of parameters in Model 37 was quite high, however. Some of these parameters were ‘second-level’ parameters, which were included to improve the properties of parameter estimates and to provide interpretations for the actual parameters of the model. There was no need to estimate too many parameters for prediction models because this study aimed at predicting rather than studying the effects of different factors on the height-dbh relationship. Furthermore, the second-level parameters may cause other hurdles in the estimation phase. Thus, Models 34 and 6, with fewer parameters (six and three parameters, respectively) were selected as the final models in this study.

The development of simple and accurate models that allow forest managers to reliably determine the height of trees in a stand from dbh data is of prime importance in forest management. In this study, the two selected models not only had good statistical reliabilities, but were also easy to apply. Estimating mean tree height was required for the practical application of the models. As shown in Fig 7 (a, b), the biases for large and small values of diameter were greater than the biases for intermediate values; this may be due to a very small number of observations in smaller (d<4cm) and larger (d>40cm) trees. The equations selected (Models 34 and 6) are therefore best used within the range of values 4 cm to 40 cm. Applications beyond this range should be used cautiously and tested further.

### Uncertainty in predicting small tree height at local scale

For the smaller tree (dbh (d) is less than 15 cm), the Fig 8 showed a distinct variations in height-dbh relationships for Chinese fir plantations between the Changsha and Huitong regions. The whole provincial height-dbh models seem to overestimate the tree height in Changsha region, but underestimate the tree height in the Huitong region. However, there was no significant variation in height-dbh relationships when the dbh (d) is larger than 15 cm. Both model 6 and 34 showed the consistent results for both regions. Model form and model parameters were essential for the model applications. Even though we have demonstrated both model 6 and 34 are robust in model structure, the parameters are therefore determining factor to influence the accuracy of the models application. For example, Kearsley et al. [8] showed that above-ground carbon stocks would be overestimated by 24% if the inaccurate tree height-dbh relationships were used. Therefore, using provincial-based tree height-dbh for predicting small tree height at local scale may result in larger uncertainties.

## Conclusions

In this study, 39 height-dbh models were calibrated and tested on trees in Chinese-fir plantations between 6 and 53 years old in subtropical China. Model selection was based on goodness of fit, precision and practical application. The results showed that composite models that included additional stand variables improved model performance. The best predictions of height were obtained using nonlinear composite Model 34 and linear composite Model 6, which used six and three parameters respectively; these were recommended for Chinese-fir plantations with a dbh range between 4 cm and 40 cm. The inclusion of the quadratic mean dbh (Dq) and mean height (Hm) as independent variables in the height-dbh equations appears to be necessary to achieve acceptable predictions. It is clear that quadratic dbh (Dq) and mean height (Hm) influence the height-dbh relationship; and furthermore, the formula Hm = 11.707×ln(Dq)-18.032 can be used to predict Hm from Dq. Although stand age was not the most important variable in the height-dbh relationship, tree height increased with tree age within the same diameter class. And the stand density did not effect tree growth in this study. The local climate at different sites also influenced the prediction of tree height; therefore there were some differences in parameter estimates for different ages and sites. The inappropriate application of provincial or regional height-dbh models to different ecoregions can produce significant errors for estimating local tree height and volumes.

The method and the recommended models developed in this study were statistically reliable for applications in growth and yield estimation and management planning for Chinese-fir plantations in central south of China. The models could be extended to other regions and to other tree species only following verification.
